# Novel kinase 1 regulates CD8+T cells as a potential therapeutic mechanism for idiopathic pulmonary fibrosis

**DOI:** 10.7150/ijms.93510

**Published:** 2024-04-22

**Authors:** Zhen-Yuan Tan, Yuan Lou, Yu-Cui Qin, Wei Lin, Bin-Bin Liang, Suren R. Sooranna, Yi-Li Ma, Su-Fang Zhou

**Affiliations:** 1 School of Basic Medicine, Guangxi Medical University, Nanning, Guangxi, 530021, China.; 2 The First Affiliated Hospital of Guangxi University of Chinese Medicine, Nanning, Guangxi 530021, China.; 3 Department of Metabolism, Digestion and Reproduction Faculty of Medicine Imperial College London Chelsea & Westminster Hospital, London SW10 9NH, UK.; 4 Life Science and Clinical Research Center, Youjiang Medical University for Nationalities,18 Zhongshan Road II, Baise 533000, Guangxi, China.

**Keywords:** Idiopathic pulmonary fibrosis, bioinformatics, immune landscape, NUAK1, CD8+T

## Abstract

Idiopathic pulmonary fibrosis (IPF) is a rare, chronic and progressively worsening lung disease that poses a significant threat to patient prognosis, with a mortality rate exceeding that of some common malignancies. Effective methods for early diagnosis and treatment remain for this condition are elusive. In our study, we used the GEO database to access second-generation sequencing data and associated clinical information from IPF patients. By utilizing bioinformatics techniques, we identified crucial disease-related genes and their biological functions, and characterized their expression patterns. Furthermore, we mapped out the immune landscape of IPF, which revealed potential roles for novel kinase 1 and CD8+T cells in disease progression and outcome. These findings can aid the development of new strategies for the clinical diagnosis and treatment of IPF.

## Introduction

Idiopathic pulmonary fibrosis (IPF), also referred to as fibrosing alveolitis, is a rare but severe chronic lung condition characterized by the gradual formation of scars within the lung tissue 1, 2. This scarring impairs the normal diastolic and systolic functions of the lungs, affecting the small air sacs and ultimately leading to serious complications such as pulmonary hypertension, embolism, respiratory failure and even death 3. Despite its rarity, IPF carries a higher mortality rate than some common lung tumors 4, 5.

Currently, early and accurate diagnosis of IPF remains a significant clinical challenge. While early detection and continuous intervention can potentially slow the disease's progression and improve long-term outcomes 6, 7, existing diagnostic strategies that rely on imaging and disease triggers often provide unspecific and inaccurate classification, especially when distinguishing IPF from other forms of interstitial pneumonia 1, 8.

The treatment for IPF is also limited, with most approaches focusing on managing symptoms and providing supportive care rather than addressing the underlying cause. This can impose a significant burden on both patients and their families. Recent studies examining bronchial lavage fluid have suggested a close association between T cells and the development and progression of IPF 9. This finding highlights the potential role of the immune system, particularly innate and adaptive immune processes, in coordinating fibrotic responses and influencing patient prognosis. It has been suggested that exploring these associations may offer new insights and potential solutions to the current clinical challenges surrounding IPF 10, 11.

Based on these findings, we downloaded the gene expression and clinical data related to IPF patients from the GEO database and used the CIBERSORT algorithm to estimate the relative fraction of T cell subtypes in the samples. We then determined two core genes most related to the progression of IPF and the accompanying changes in T cells. After verification of our findings in rats, we finally identified the novel (nua) kinase 1 (NUAK1), also known as 5' adenosine monophosphate-activated protein kinase-related kinase 5 (ARK5), as a key gene with potential diagnostic and therapeutic value for IPF.

## Materials and Methods

### Databases

The GSE28042 and GSE70866 datasets were retrieved from the gene expression omnibus (GEO) database in MINiML format. Differential expression of mRNA was analyzed using the limma package in R software version 3.40.2. To correct for false positives, the adjusted P values were computed within the GEO. mRNAs with adjusted P values less than 0.05 and log2 fold changes greater than 1.3 or less than -1.3 were considered differentially expressed genes (DEGs). Using the ggplot2 package in R, normalized maps and volcano plots were generated for the samples. Expression heatmaps were visualized using the Complex Heatmap package in R.

### Functional enrichment of differential genes

Functional enrichment analysis was conducted in order to further validate the potential functions of target genes. Gene Ontology (GO) was used for annotating genes based on their molecular function (MF), biological pathway (BP), and cell component (CC). Additionally, the Kyoto Encyclopedia of Genes and Genomes (KEGG) enrichment analysis was used to determine advanced genomic function information. The ClusterProfiler package in R was used to determine the role of target genes with respect to the GO functions of potential mRNAs and KEGG pathway enrichment.

### Immunoassay

The CIBERSORT (https://cibersortx.stanford.edu/) algorithm was used to evaluate the GSE dataset for samples with different relative fractions of T cell subtypes.

### Weighted gene co-expression network analysis (WGCNA)

The R package WGCNA was used to generate a comprehensive gene network using data from the GSE28042 dataset. This identified multiple modules of co-expressed genes that were strongly associated with T cell subtypes. To establish a robust and biologically meaningful gene co-expression network, a power of β=8.5 and a scale-free R-squared value of 0.82 were chosen as the threshold parameters, ensuring a signed and scale-free network representation.

### Screening of hub genes

A Venn diagram was used to visualize the overlap between the identified modules and the DEGs. Subsequently, LASSO logistic regression and Random Forest methods were utilized to identify and select the most significant hub genes from the dataset.

### Gene correlation analysis

Spearman correlation analysis was used to evaluate the correlation between T cell levels and the hub genes found in the GSE28042 and GSE70866 datasets.

### GSEA analysis

The KEGG pathway enrichment analysis was performed for gene set enrichment analysis (GSEA). The "enrichplot" R package was used on the GSEA hub genes and p < 0.05 was considered statistically significant.

### Protein-protein interaction (PPI) network

GeneMANIA (http://genemania.org/), which is an online tool for studying the genes which are related and similar to the target genes, was used to establish the PPI networks associated with the hub genes.

### Statistical analysis

The Wilcoxon rank-sum test was utilized to determine the statistical differences between two groups. Kaplan-Meier analysis provided a visual representation of survival disparities, which were subsequently assessed using the log-rank test. Spearman's correlation coefficient was calculated to assess the association between RR subunits and the immune microenvironment. All statistical analysis were performed using R software (version 4.1.3), and statistical significance was defined as a p-value less than 0.05.

### Experimental animals used and drug preparation

Nineteen male SD SPF grade rats, aged 6-8 weeks and weighing approximately 200±20g, were sourced from the Experimental Animal Center of Guangxi Medical University, which hold the appropriate licenses for the use of animals in research. The rats were housed under controlled conditions and maintained at 20-25°C with a relative humidity of 40-70%, and a regular day-night cycle with 12 hours of lighting. The Animal Ethics Committee of the Guangxi Medical University approved this study (No. 202201199).

The therapeutic drug used was a Guizhi plus *Houpo Xingzi* decoction (GHXD), which included the Chinese medicinal materials cinnamon twigs, licorice, ginger, peony, jujubes, *Magnolia officinalis* and almonds. The rats were randomly assigned to two groups: one group (n=15) was exposed to dust contamination using silica as the inducer, and the other group served as the non-dust-exposed control group (n=4). After dust exposure, the contaminated rats were further randomized into three subgroups each of which had 5 animals: the model group, the pirfenidone treatment group and the GHXD treatment group.

The daily dose concentration given to the rats was calculated to be 0.8 g/kg based on the equivalent dose coefficient conversion algorithm. In preliminary experiments it was found that after 35 days of dust exposure provided a good modeling performance. Starting from the second day of modeling, the drug was administered continuously for 35 days to achieve the optimal modeling effect under our experimental conditions. The model and control groups were given corresponding amounts of physiological salts during the study period.

### Lung histopathology

Lung tissues were fixed and routinely embedded in paraffin to prepare 4 μm tissue sections. These were stained with hematoxylin-eosin (HE) and Masson stains and observed and photographed using a microscope (BX43, Japan).

### Lung tissue immunohistochemistry

The silicosis-related genes identified through network pharmacology were further analyzed using immunohistochemistry. Initially, paraffin sections were rehydrated with xylene solutions (I, II and III) for 10 minutes each, followed by graded ethanol for 2 minutes each time. After washing with distilled water, antigen retrieval was performed. Subsequently, the sections were washed with PBS and the nonspecific antigen sites were blocked. Then, they were incubated with primary antibodies (CD8 monoclonal rabbit antibody (FSG062011A, Novus Biochemical, UK) and ARK5 rabbit polyclonal antibody (BB09161803, Beijing Boosten Biotechnology Co. Ltd., China) for 2 hours at room temperature. After washing with PBS, the sections were incubated with a secondary antibody conjugated to horseradish peroxidase (MaxVision TM HRP Polymer anti Mouse/Rabbit IHC Kit, 220901S407n, MXB Biotechnology, Fuzhou, China) for 40 minutes at room temperature. Following three washes with PBS, a DAB color development kit was used. The stained sections were observed and photographed under a microscope. Image J software was used to quantify the positive areas between two different sections, and GraphPad Prism was used for statistical analysis of the data.

## Results

### Data processing

**Figure 1:**
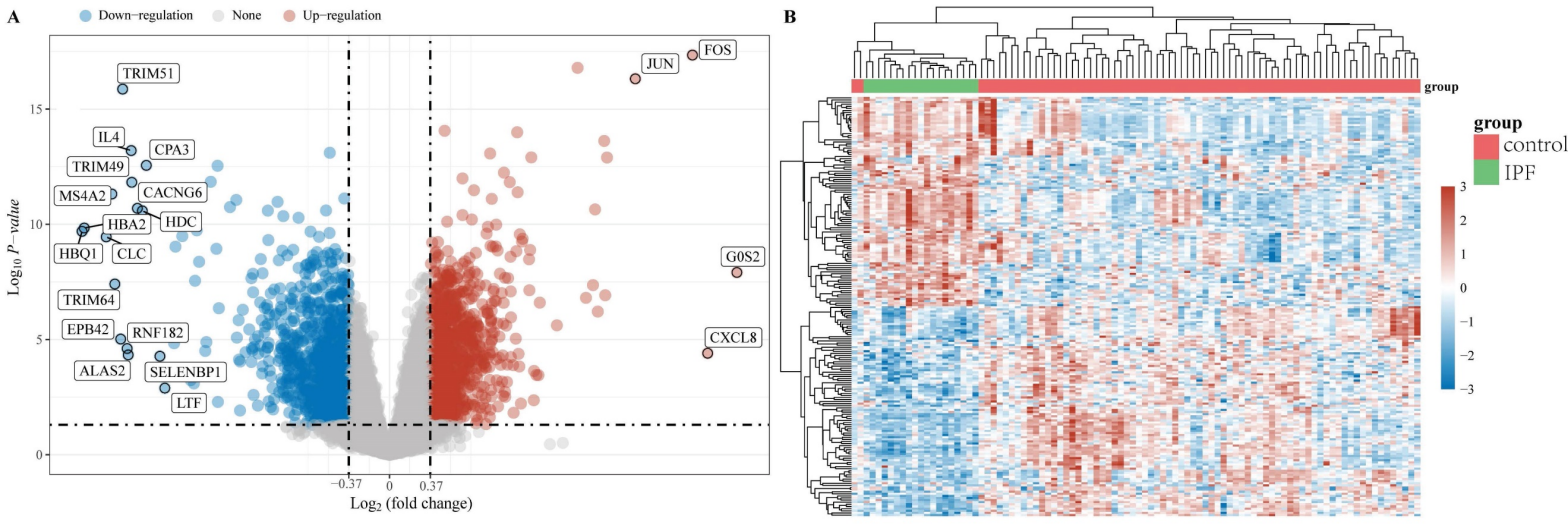
Data preprocessing. (A) A differential gene expression volcano plot is displayed using fold change and corrected P values. (B) A differential gene expression heatmap in which the different colors represent the expression trends in different tissues.

The "limma" package was utilized to investigate the mRNA differential expression in the GSE28042 dataset. A screening threshold was established where mRNAs with an adjusted P-value of less than 0.05 and a log2 fold change greater than 1.3 or less than -1.3 were considered to be significantly and differentially expressed. Using these criteria, 2222 DEGs were identified, comprising 1093 up-regulated and 1129 down-regulated genes and are shown in a volcano plot (Figure 1A). Furthermore, a heatmap was generated to visualize the top 50 most significantly up-regulated and down-regulated genes based on their differential expression levels (Figure 1B).

### Functional enrichment analysis of DEGs

**Figure 2:**
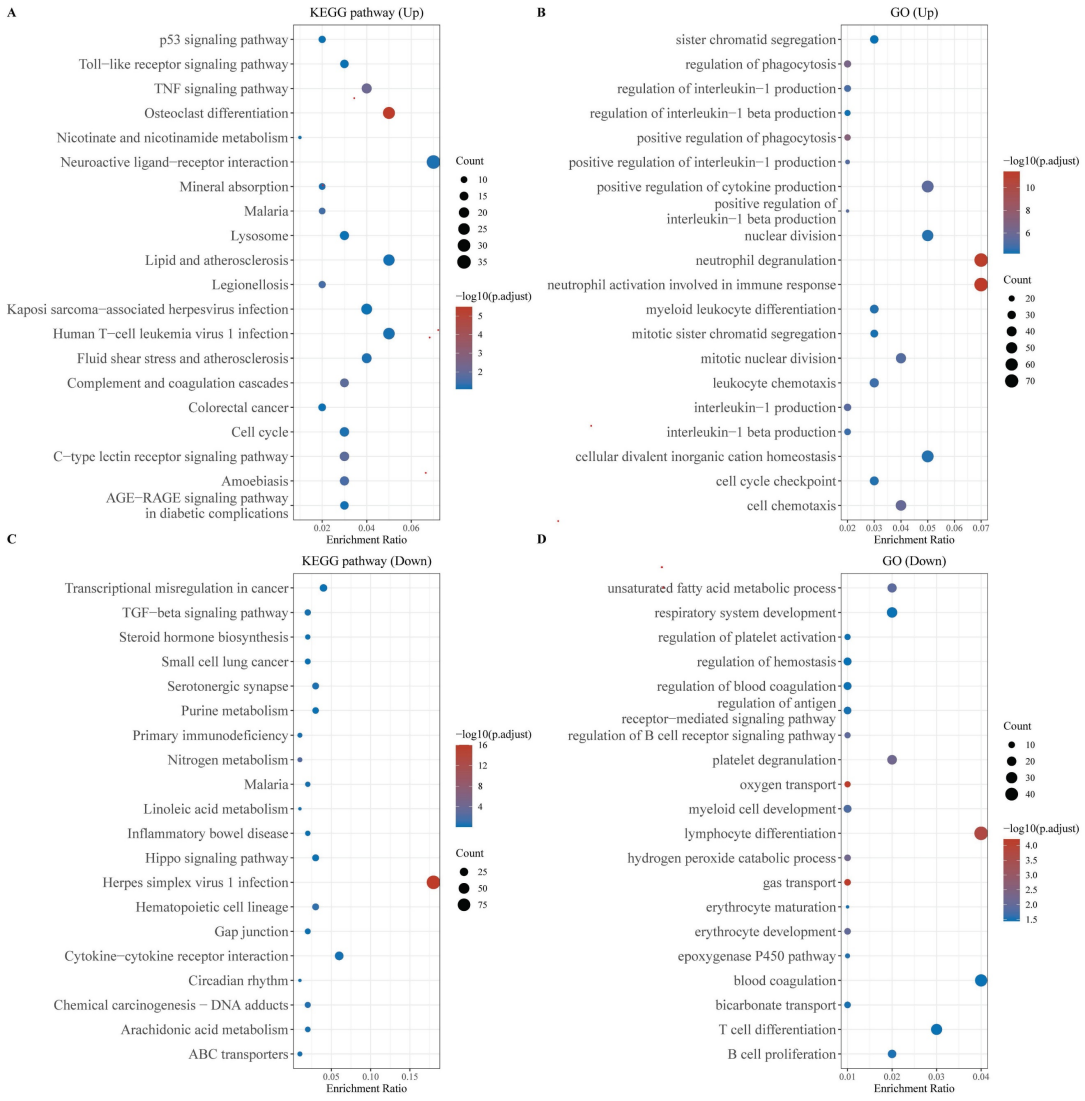
Functional enrichment analysis of DEGs. (A) KEGG enrichment analysis of the up-regulated DEGs, (B) KEGG enrichment analysis of down-regulated DEGs, (C) GO enrichment analysis of up-regulated DEGs, and (D) GO enrichment analysis of down-regulated DEGs. The larger the value, the smaller the FDR value. The size of the circles represents the number of enriched genes. The larger the circle size, the larger the number. In the enrichment results, p < 0.05 represents a statistically significant difference.

We performed functional enrichment analysis separately for the up-regulated and down-regulated DEGs. KEGG pathway analysis revealed that the up-regulated genes were primarily enriched in the P53 and TNF signaling pathways, as well as in cellular processes related to the cell cycle and malaria. Conversely, the down-regulated genes exhibited significant enrichment in pathways associated with Herpes simplex virus 1 infection and cytokine-cytokine receptor interactions (Figures 2A, 2B). GO analysis of the up-regulated genes highlighted their involvement in neutrophil degranulation, neutrophil activation during the immune response, and the maintenance of cellular divalent inorganic cation homeostasis (Figure 2C). The down-regulated genes were predominantly enriched in processes related to lymphocyte differentiation, oxygen transport, and gas transport (Figure 2D).

### WGCNA network construction

**Figure 3:**
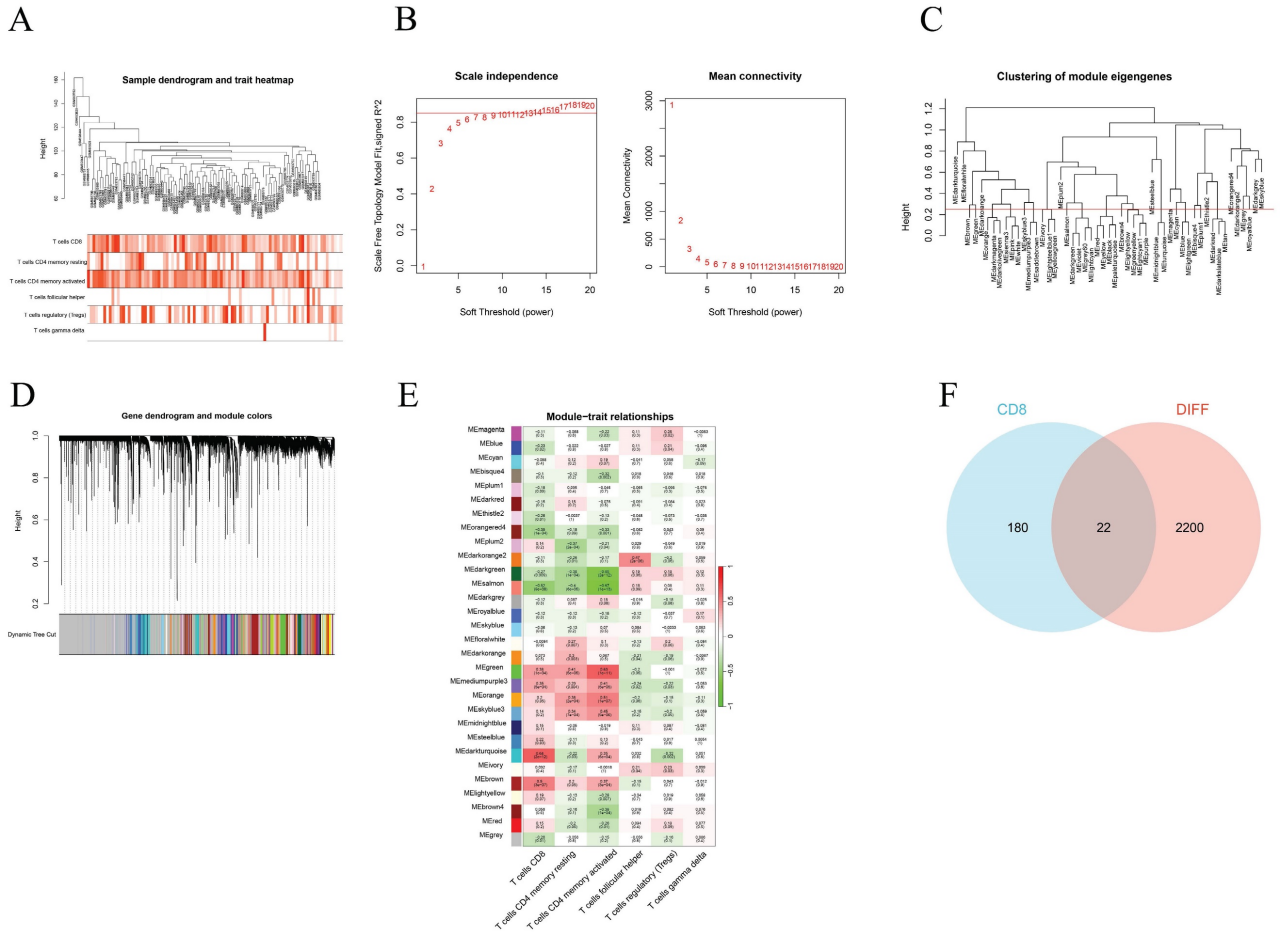
Construction of the WGCNA co-expression network. (A) Sample clustering dendrograms with tree leaves corresponding to the individual samples. (B) Soft threshold β = 6 and scale-free topological fit index (R2). (C) Clustered dendrograms were cut at a height of 0.25 in order to detect and combine all the similar modules. (D) Shows the original and combined modules obtained under the clustering tree. (E) A heatmap of module-trait correlations. (F) The turquoise module genes and DEGs when analyzed and displayed as a Venn diagram.

We used WGCNA to screen the genes associated with different T-cell subtypes. Firstly, sample clustering dendrograms for correlations between samples and different T-cell subtypes were constructed. The R package "pickSoftThreshold" was then used to calculate the soft threshold power β, and 30 modules were subsequently generated through network construction (Figure 3).

### Module gene screening and functional enrichment analysis

Through the above WGCNA analysis, it was found that the dark turquoise colored module genes were most correlated with CD8+ T cells (relativity =0.64, P=2e-12) (Figure 3E). A follow-up analysis was conducted for the 202 genes in this module by intersecting them with the DEGs found and this showed 22 common genes (Figure 3F).

### Screening of hub genes

**Figure 4:**
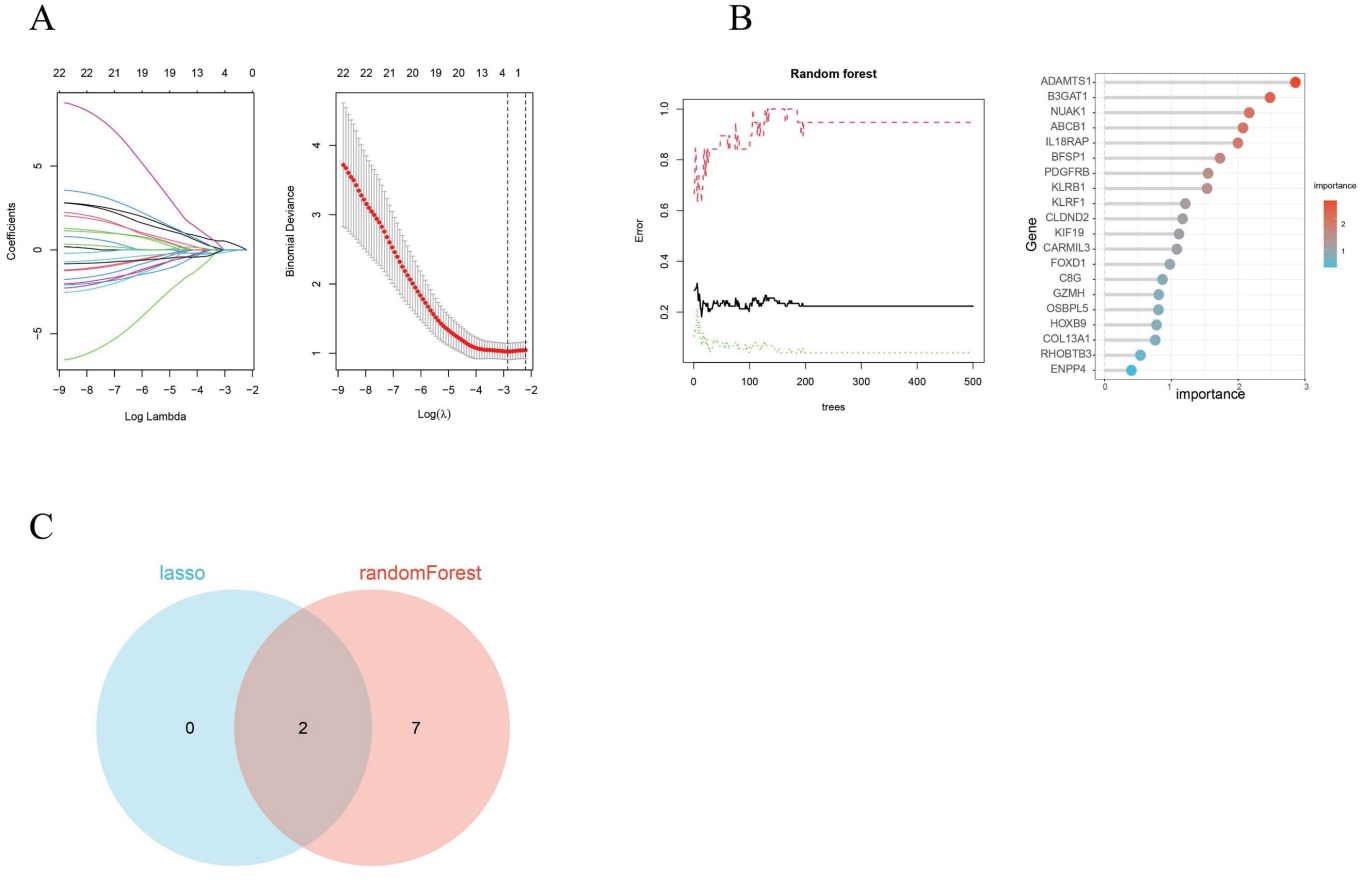
Hub gene selection. (A) Adjustment of feature selection in the least absolute shrinkage and selection operator model (LASSO). (B) The Random Forest error rate versus the number of classification trees and the top 20 DEGs found. (C) A LASSO- and Random Forest-generated Venn diagram of the genes screened.

By utilizing both LASSO regression and Random Forest algorithm analyses, we identified 2 and 9 hub genes, respectively (Figures 4A, 4B). A Venn diagram was used to visualize the overlap between these two sets of genes and this showed 2 common genes, IL18RAP and NUAK1 (Figure 4C).

### Identification of the hub genes

**Figure 5:**
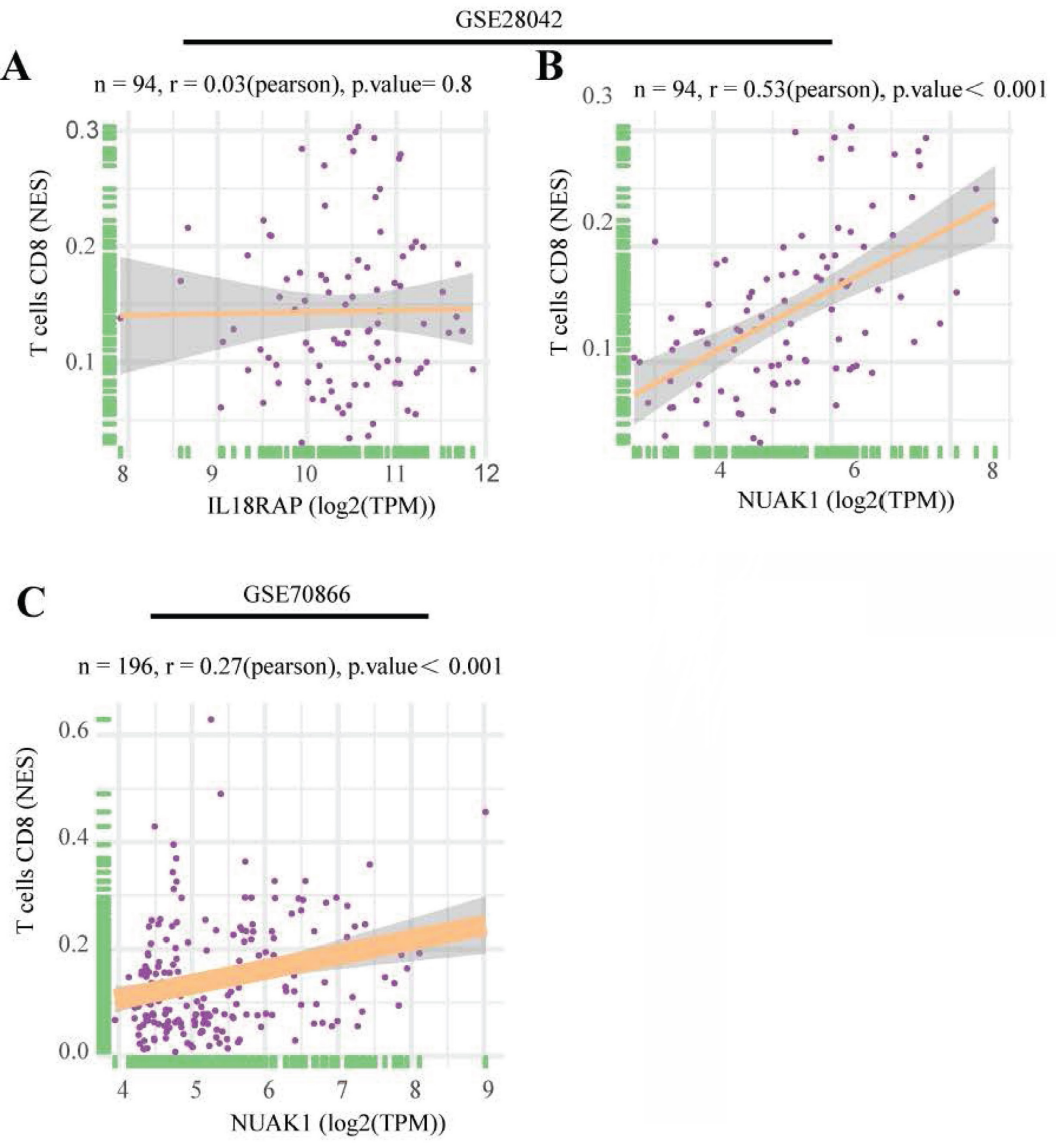
Scatter plots of hub gene expression versus CD8+ T cells infiltration levels from three different datasets: (A-B) GSE28042. (C) GSE70866.

**Figure 6:**
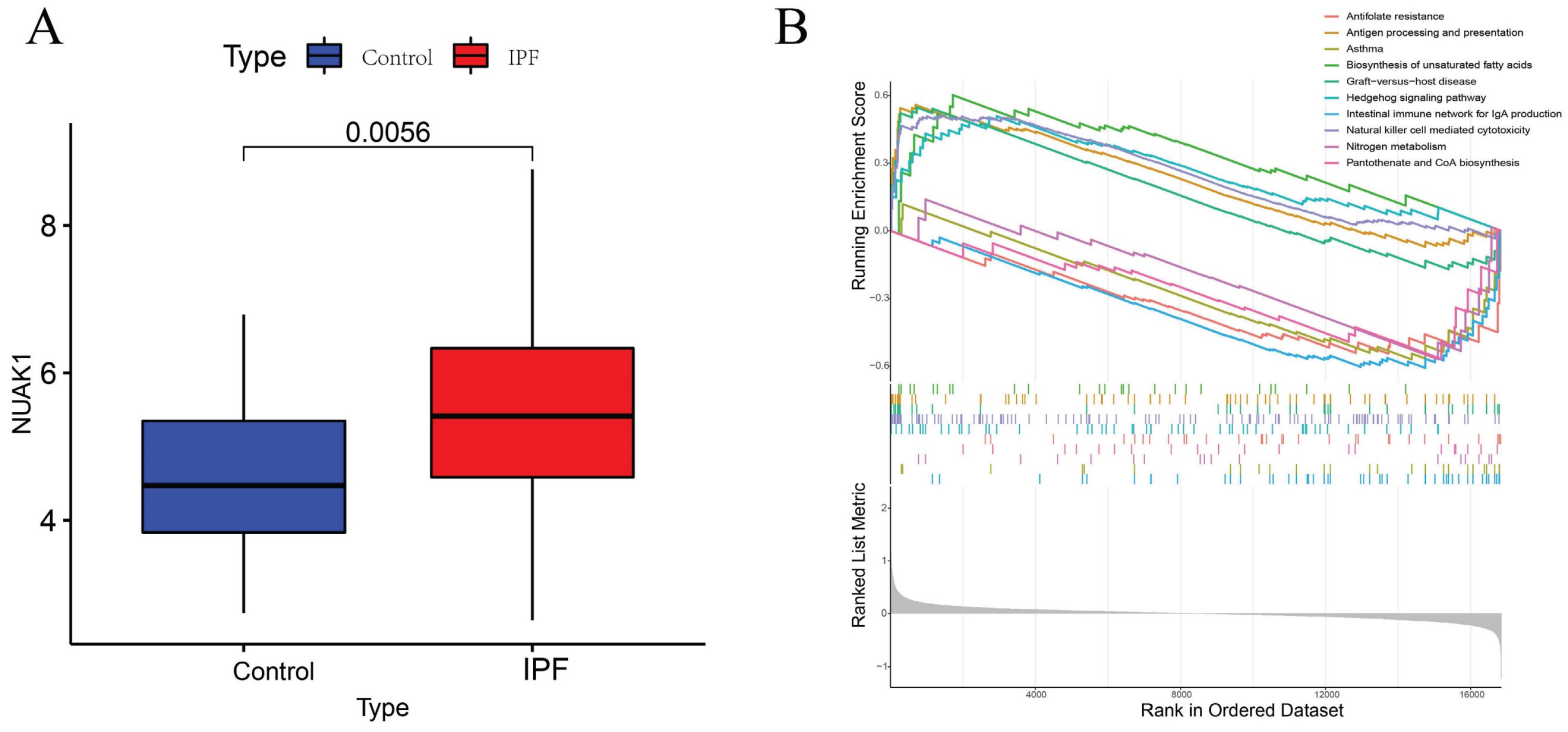
GSEA analysis of hub genes. (A) Expression levels of NUAK1 in controls and IPF patients. (B) GSEA analysis of NUAK1 expression.

To further explore the significance of these hub genes, we used the GSE28042 and GSE70866 datasets to assess their correlation with CD8+ T cell infiltration levels. Analysis of the GSE28042 dataset revealed that both IL18RAP and NUAK1 exhibited a positive correlation with CD8+ T cells, but the correlation for IL18RAP was minimal (r=0.03, p=0.8) compared to that of NUAK1 (r=0.53, p<0.01; Figures 5A, 5B). By using the GSE70866 dataset, IL18RAP was excluded from subsequent studies, as only NUAK1 showed a significant positive correlation with CD8+ T cells (r=0.27, p<0.01) (Figure 5C). A boxplot was utilized to visualize the expression of NUAK1 in the GSE28042 dataset, demonstrating its elevated expression in IPF (Figure 6A).

### GSEA analysis

According to GSEA analysis, NUAK1 was enriched in 29 different biochemical processes (Figure 6B), including human immunodeficiency virus 1 infection, transcriptional dysregulation in cancer, Th1 and Th2 cell differentiation and PD-L1 expression. The T cell receptor and Hippo signaling pathways as well as the PD-1 checkpoint pathway in cancer were also implicated.

### PPI network and enrichment analysis

The GeneMANIA database was utilized to assess the NUAK1-related genes and these were subjected to functional enrichment analysis. The 20 most relevant genes were found to include PRKAG1, LATS1, PPP1CB, USP9X and CDKN1A. GO enrichment analysis showed cellular response to starvation, protein kinase complex, transferase complex, transferring phosphorus-containing groups, protein serine/threonine kinase activity, transferase complex, transferring phosphorus-containing groups, protein serine/threonine kinase activity and protein serine kinase activity were primarily associated with IPF. KEGG enrichment analysis was enriched mainly in the oxytocin signaling pathway, proteoglycans in cancer, insulin signaling pathway and regulation of actin cytoskeleton.

### HE and Masson staining to observe the pathological changes of lung tissues

**Figure 8:**
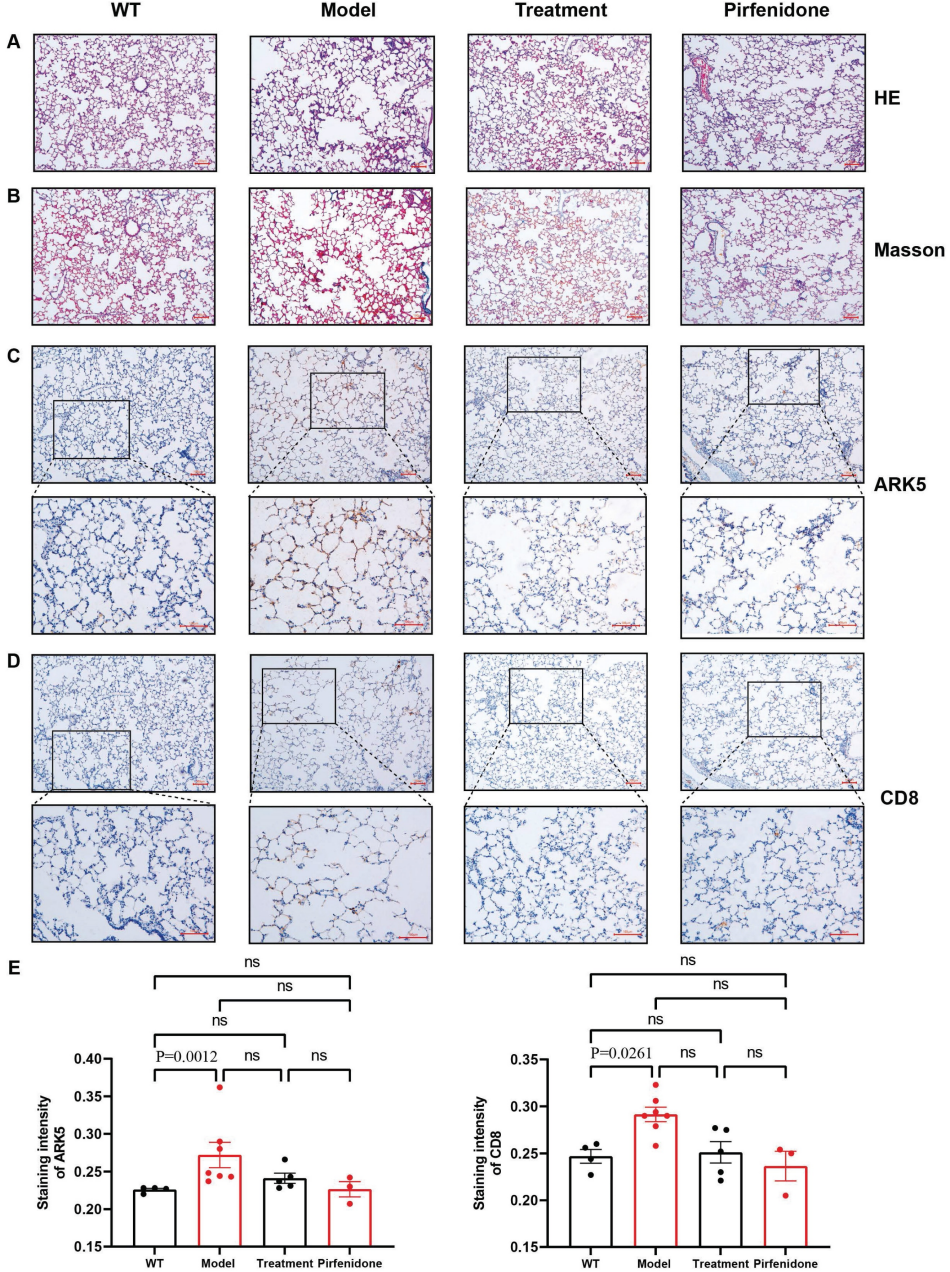
Statistical analysis of pathological and immunohistochemical staining. A: HE staining (100x); B: Masson staining (100x); C: Immunohistochemical staining of NUAK1 using a rabbit polyclonal antibody to ARK5; D: Immunohistochemical staining of CD8 using a rabbit monoclonal antibody; Scale bar = 100µm E: Differences in staining of NUAK1 and CD8 between the different experimental groups. p < 0.0261 and 0.0012, respectively, were for the differences in the positive areas between the WT and model groups; n= 4 for the control group and n=5 for each of the other 3 groups.

HE and Masson staining of rat lung tissue sections showed that the control group had a complete and clear alveolar structure and normal alveolar intervals, and no obvious pathological changes were found. However, the alveolar structures of rats in each of the dust-contaminated group were seriously damaged and disordered with the appearance of obvious alveolar intervals. The alveolar tissue became wider and larger, and collagen fiber deposition was obvious. After drug interventions, the alveolar tissue structures were relatively complete, with less collagen accumulation although some abnormalities remained (Figures 8A, 8B).

### Immunohistochemistry of NUAK1 and CD8 in normal and pulmonary fibrotic lung tissues

Network pharmacology showed that the NUAK1 gene may be involved in the differentiation process of T helper cell subsets and thus participate in the occurrence and development of pulmonary fibrosis. It was found that the expression levels of NUAK1 in normal tissues were weak but it was strongly expressed a in pulmonary fibrotic tissues. This trend was mimicked by the expression of CD8 in the T helper cell subsets. The positive areas and staining intensities in the IHC results were analyzed by the ImageJ plugin IHC Profiler, and statistical analyses were carried out using GraphPad Prism 12. The results showed that the expression levels of NUAK1 and CD8 in normal lung tissue were significantly lower than those in pulmonary fibrosis lung tissues (P<0.05 in both cases; Figures 8C-8E).

## Discussion

IPF, a potentially severe and progressive lung disease, remains a diagnostic and therapeutic challenge for clinicians, and its underlying pathogenesis remains unclear. Central to its development and progression are the fibrotic and scarring processes triggered by inflammation. When the damage is significant or the person involved is exposed to persistent harmful environments, this can lead to local and systemic chronic inflammatory reactions as well as sustained damage to capillary endothelial cells and alveolar epithelial cells. These can promote the migration, proliferation and transformation of lung fibroblasts, releasing extracellular matrix (ECM) factors, thereby leading to the development of pulmonary fibrosis 13-15. Initially a role for T cells in IPF via the inflammatory cytokine, IL-6, was implicated. Moore and Herzog (2016) subsequently showed that inhalation of silica (SiO2) led to the deletion of FoxP3 which resulted in increased accumulation of effector T cells and a IL-4-mediated fibrotic response 16-18. In this study we used bioinformatics and machine learning-driven analysis of genetic data from IPF patients from the GEO database to identify potential links between IPF and T cells. We identified NUAK1, which is associated with CD8+T cells, as a promising diagnostic biomarker and therapeutic target for IPF.

NUAK1, also known as NUAK family SNF1-like kinase 1 and ARK5, primarily functions in regulating the cell cycle and cell adhesion. Its significance extends to malignancy, with numerous studies highlighting its crucial role in tumor invasion, growth, and metastasis 19-21. Zhang et al. (2022) showed that NUAK1, can also promote pro-fibrotic YAP and TGF-β/SMAD signaling, ultimately leading to organ scarring, which was confirmed using mouse models of kidney, lung, and liver fibrosis 22. Yang et al. (2022) found that NUAK1 induces epithelial mesenchymal transition (EMT) in liver cells and promotes the secretion of inflammatory factors. These can enhance the activation of hematopoietic stem cells, promoting liver fibrosis through the synergistic effect between hematopoietic stem cells and liver cells 23. However, in the kidneys, it has been shown that silencing of NUAK1 reduces the expression of TGF-β1 and reversing fibrosis of renal tubular cells, thus alleviating the symptoms of renal fibrosis 24. Our study also suggests that NUAK1 may play a pivotal role in pulmonary fibrosis. In addition, Morito et al. showed an upregulation of NUAK1 in T cell lymphoma, suggesting its role as a c-Maf excessive factor 25. Our enrichment analysis indicated that this gene might be involved in the differentiation of T helper cell subsets.

T cells are pivotal in the fibrotic processes in various organs. For instance, they contribute to wound healing and tissue repair following liver injury, while exhibiting a protective role in kidney fibrosis 9, 26. Recently, several studies have highlighted the role of T cells in IPF. Li et al. (2021) discovered that CD247, primarily expressed by T and NK cells in the lung, were involved in T cell maturation and differentiation, suggesting it has potential as a biomarker for IPF severity and prognosis 27. Habiel et al. (2019) observed a significant increase in CD8+ CD28null T cells in IPF patient lung cell suspensions 28. Our study found a unique role of CD8+ T cells in IPF, and these were present in bronchial lavage fluid and tissue biopsies of IPF patients 29-31. Wang et al. (2022) reported impaired proliferation of CD8+ T cells in response to donor antigens among IPF patients prior to lung transplantation 32, though the mechanisms remain elusive. We identified NUAK1 as a potential gene target strongly associated with CD8+ T cell infiltration in IPF patients and it was a driver of fibrosis.

We analyzed the GSE28042 dataset and compared mRNA expression differences between patients with IPF and a healthy control group. 1,093 genes were upregulated and 1,129 were downregulated. The upregulated genes were primarily enriched in the P53 signaling and TNF signaling pathways, indicating their association with critical biological processes such as cell apoptosis, cell survival regulation and the inflammatory response. Simultaneously, the downregulated genes were enriched in pathways related to Herpes simplex virus 1 infection and the interactions between cytokines and their receptors. This suggested their involvement in weakening of the immune and defense mechanisms. GO functional enrichment analysis showed that upregulated genes were concentrated in functions such as neutrophil degranulation and activation. The downregulated genes were significantly enriched in lymphocyte differentiation, oxygen transport and gas transport, suggesting changes in immune function during disease progression and reduced efficiency of gas exchange.

WGCNA identified two core genes, IL18RAP and NUAK1, which are associated with the pathological process of IPF. NUAK1 exhibited a strong significant positive correlation with CD8+ T cells, and this was validated with the GSE70886 dataset. The correlation with IL18RAP was weaker and not statistically significant and it was excluded from subsequent studies. However, as this association was based only on the GEO database, this was limited and subjected to bias. Therefore, we conducted animal experiments to validate the effects of NUAK1 gene in animal models. We tested the treatment of a type of traditional Chinese medicine which has been used to to slow down and improve the progression of IPF. The classic Chinese medical text "Shanghan Lun" records that the formula GHXD was primarily used to treat conditions such as "unresolved exterior syndrome with slight wheezing after sweating". It has been utilized by successive generations of physicians for dispelling qi, stabilizing wheezing and relieving cough symptoms. The symptoms of silicosis-induced fibrosis align closely with those described by GHXD, indicating its compatibility with the specific clinical patterns. Moreover, the extensive clinical application of GHXD, in our preliminary experiments it was shown to have inhibitory effects on silicosis-induced IPF, prompted us to use it as a potential treatment.

We initially focused on silicosis-induced IPF and conducted preliminary tests using this experimental model with suspensions of SiO2 which were used as the mold-forming substance. The optimal dosage of 50 mg/mL of SiO2 suspension 33-34 was used as it had the most favorable molding effects and low animal mortality. Regarding the selection of model animals, C57BL/6 mice and SD rats were used and the latter was chosen due to having wider tracheas for convenient and accurate administration of the mold-forming agent.

*In vivo* experimental results showed that in rat models of IPF, the expression levels of NUAK1 and CD8+ T cells were significantly higher compared to healthy control rats. This finding was in agreement with the results obtained from network pharmacology; this outcome verified the critical role of NUAK1 in regulating the functionality of CD8+ T cells and its impact on the onset and progression of IPF and that this kinase is a potential therapeutic target for the treatment of IPF.

## Conclusions

Our study showed a central and unique role of NUAK1 in IPF by using the patient sample data from the GEO database. The findings were verified by conducting experiments in a rat model of this condition. This study has provided a potential new method for the early accurate diagnosis of IPF. In addition, the efficacy of a treatment regimen for this condition was explored.

## Figures and Tables

**Figure 7:**
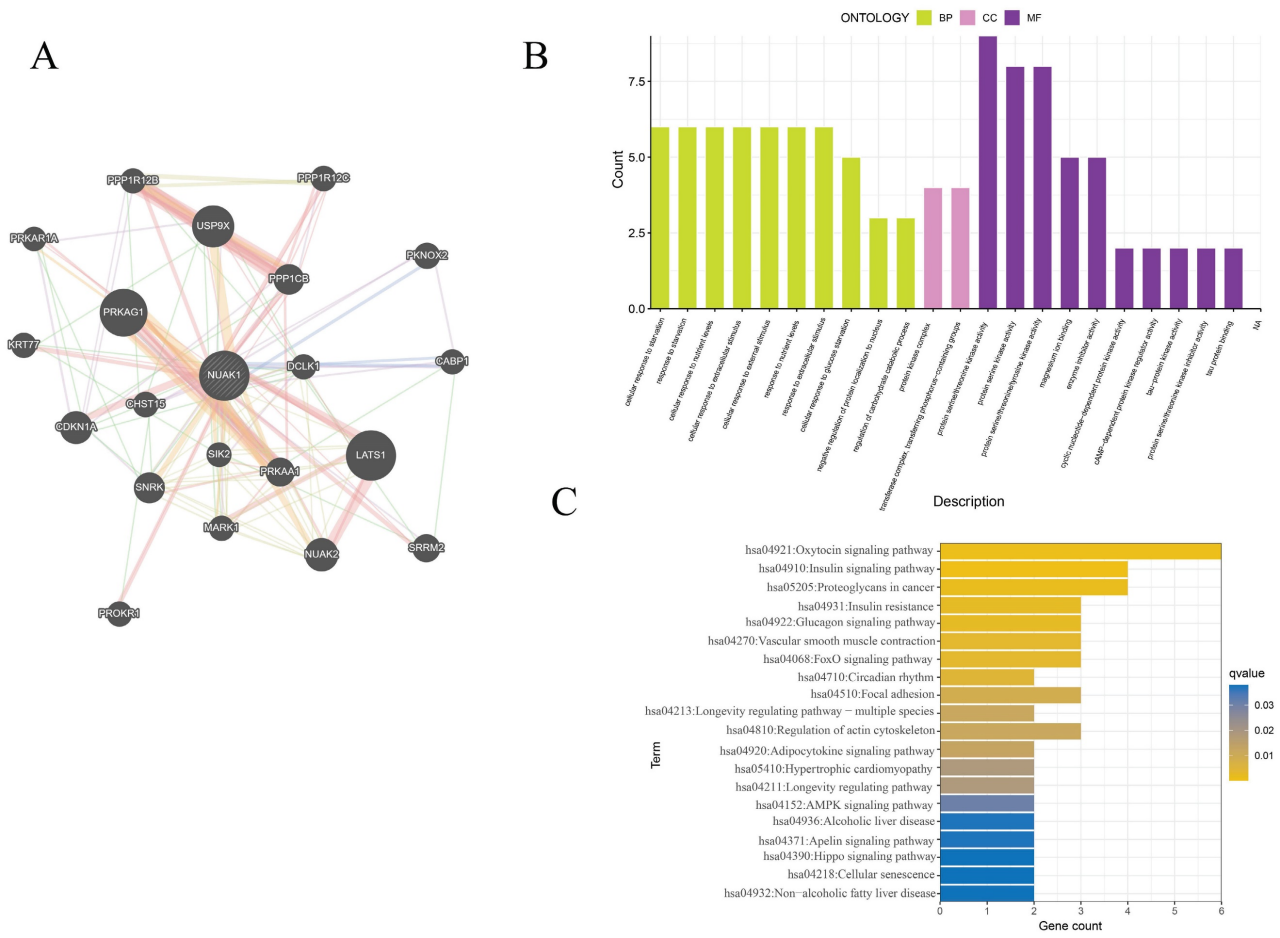
PPI network and enrichment analysis of hub genes. (A) The PPI networks associated with NUAK1 expression. (B) GO analysis of genes associated with the PPI networks. (C) KEGG analysis of genes associated with the PPI networks.
